# Microcystic adnexal carcinoma of the eyelid and orbit: A case report and review of literature

**DOI:** 10.1097/MD.0000000000034709

**Published:** 2023-08-11

**Authors:** Shiwei Huang, Yang Xia, Yueyang Zhu, Zhiyuan Ren, Yaru Dong

**Affiliations:** a Department of Ophthalmology, The Second Hospital of Jilin University, Changchun, Jilin, China; b Department of Pathology, The Second Hospital of Jilin University, Changchun, Jilin, China; c Department of Mechanical Engineering, University of Illinois Urbana Champaign, Champaign, IL, USA.

**Keywords:** eyelid, microcystic adnexal carcinoma, Mohs micrographic surgery, orbit

## Abstract

Microcystic adnexal carcinoma (MAC), a rare and low-grade malignant skin tumor, is characterized by a high rate of misdiagnosis and a preponderance for local recurrence, but seldom seen nodal or distant metastasis. Although MAC typically occurs almost in the head and neck region, primary eyelid or orbital MAC is very rare. To explore the unique characteristics of the eyelid and orbital MAC, we reviewed the relevant literature. Based on its distinctive anatomical location and the aggressive behavior, eyelid or orbital MAC not only exhibit a high rate of misdiagnosis and local recurrence, but also lead to serious complications such as disfigurement after orbital exenteration, paranasal sinuses or intracranial invasion, even death. Misdiagnosis of MAC commonly result from its rarity and nonspecific clinical and histopathological presentation. To reduce or avoid misdiagnosis, it is important to increase awareness for MAC and obtain a full-thickness biopsy specimen in histopathological analysis. Due to its extensive invasive growth pattern, MAC has a high rate of local recurrence, so complete excision with clear margins and long-term follow-up of patients with MAC are necessary. About those serious complications of the eyelid and orbital MAC, early and accurate diagnosis, complete excision is very important. Moreover, an interprofessional team consisting of ophthalmologist, otolaryngologist, neurologist, dermatologist, pathologist, radiologist is needed to evaluate and treat this disease. In summary, increasing awareness, early and accurate diagnosis, complete excision, long-term follow-up, and a multidisciplinary team is crucial for management of the eyelid and orbital MAC.

## 1. Introduction

Microcystic adnexal carcinoma (MAC), a rare and low-grade malignant skin tumor, is characterized by a high rate of misdiagnosis and a preponderance for local recurrence, but seldom seen nodal or distant metastasis.^[1]^ MAC was originally described by Goldstein in 1982,^[2]^ and it is also known as sclerosing sweat duct carcinoma, malignant syringoma, syringoid eccrine carcinoma, eccrine epithelioma, syringomatous carcinoma, sweat gland carcinoma with syringomatous features.^[3]^ Surveillance, Epidemiology and End Results database shows the yearly incidence rate of MAC is 0.52 cases per 1000,000 people, and women are affected more often than men (57.9% vs 42.1%).^[4]^

MAC is a benign appearing lesion that often presents as an asymptomatic, slow-growing, indurated, flesh-colored to yellowish nodule, plaque on the central face area, commonly the upper lip, and nasolabial folds.^[5]^ Histologically, MAC is characterized by follicular and sweat gland differentiation and overall benign features; however, it exhibits locally aggressive behavior that typically extends a few centimeters beyond the clinically visible margins, and usually infiltrates deeply into the muscle, cartilage, bone, and nerves.^[6]^ Perineural invasion was commonly seen in MAC and often leads to local recurrence. Whenever feasible, it is optimal to surgically remove the tumor entirety.^[7]^

Although MAC has been reported to occur almost exclusively in the head and neck region, primary eyelid or orbital MAC is very rare. Based on its distinctive anatomical location and the aggressive behavior, primary eyelid or orbital MAC can lead to serious complications such as disfigurement after orbital exenteration, paranasal sinuses or intracranial invasion, even death. To explore the unique characteristics of the eyelid and orbital MAC, we reviewed the relevant literature.

## 2. Methods

### 2.1. Case report

We report a patient with MAC of the eyelid who had previously experienced misdiagnosis and twice-recurrent.

### 2.2. Strategy of literature search

We conducted a comprehensive search of PubMed/Medline from inception to March 26, 2023, using the following search queries: ((MAC [MeSH Terms]) OR (Sclerosing sweat duct carcinoma [MeSH Terms]) OR (Malignant syringoma [MeSH Terms]) OR (Syringoid eccrine carcinoma [MeSH Terms]) OR (Eccrine epithelioma [MeSH Terms]) OR (Syringomatous carcinoma [MeSH Terms]) OR (Sweat gland carcinoma with syringomatous feature [MeSH Terms])) AND ((eyelid [Text Word]) OR (orbit [Text Word])). We included only English-language articles reporting on MAC located in the eyelid or orbit and reviewed the reference lists of the articles to identify additional citations.

## 3. Results

### 3.1. Case presentation

**Figure 1.**
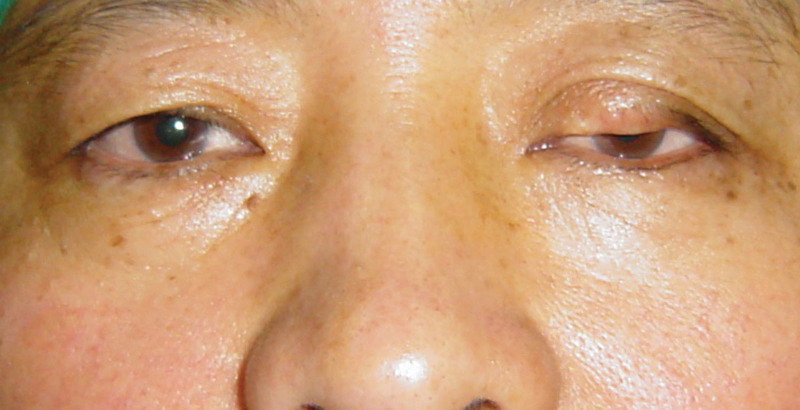
External appearance of the patient at presentation. The left upper eyelid presents ptosis, has an ill-defined, approximately 20 × 7 mm, flesh-color, indurated nodule at the central part of the eyelid. The central lid margin showed a loss of architecture with thickening, focal loss of lashes, and telangiectasia. But it had no ulceration.

A 46-year-old Chinese woman presented with a slow-growing, asymptomatic nodule that had recurred twice on her left upper eyelid over the past ten years (Fig. 1). The first occurrence was removed ten years ago at a district hospital due to occlusion of vision, and was diagnosed as a papilloma on pathology. The tumor recurred locally at the same site two months after the first operation and was excised again ten months later. Soon after, a second recurrence of the tumor appeared, which was painless and grew slowly over the past nine years. On examination, the patient had ptosis of the left upper eyelid and an ill-defined, approximately 20 × 7 mm flesh-colored nodule at the central part of the upper eyelid. The central lid margin showed thickening, focal loss of lashes, and telangiectasia, but no ulceration. No submental or cervical lymphadenopathy was observed, and the patient’s general condition was good.

Although the initial clinical impression was that the lesion was benign, the history of twice-recurrent tumor raised the possibility of malignancy. Therefore, full-thickness eyelid resection with Mohs micrographic surgery (MMS) was performed. Intraoperative frozen section suggested a benign adnexal tumor and confirmed negative margins. Reconstruction of the eyelid defects was performed immediately using an adjacent skin advancement flap and allogeneic scleral graft. Later, the permanent diagnosis of MAC was made on paraffin sections.

**Figure 2.**
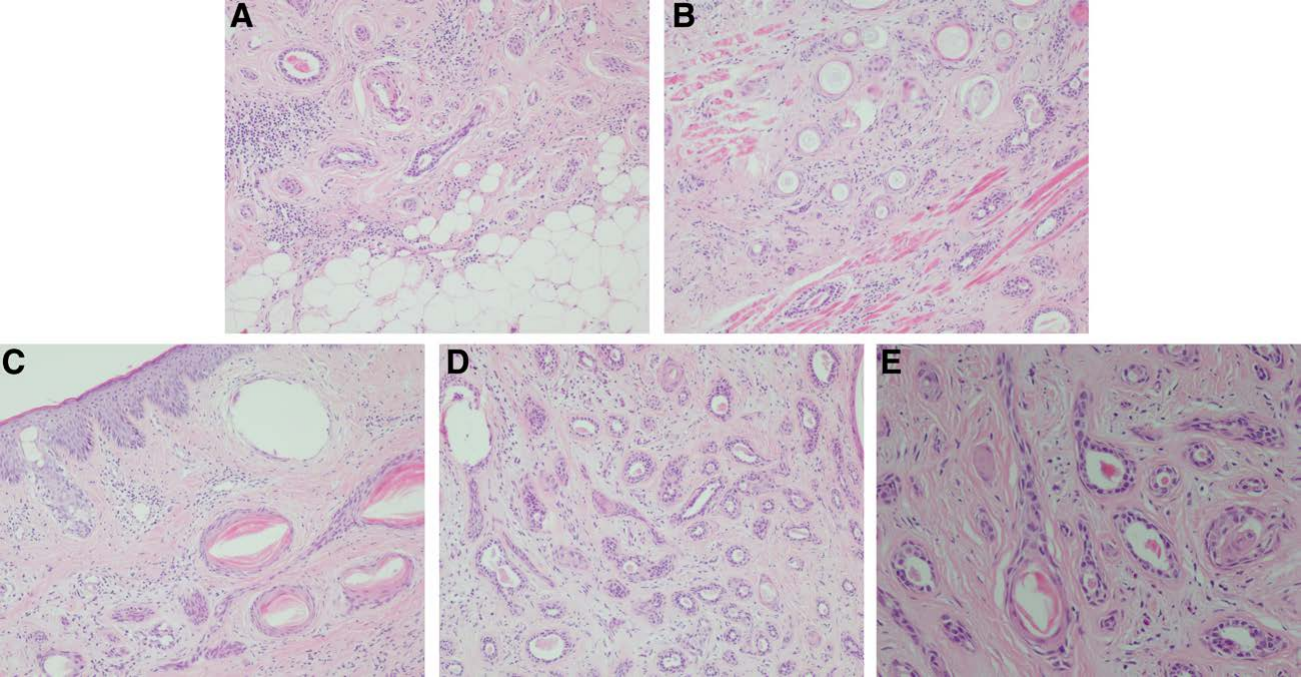
Histopathologic finding of MAC invading the deep tissues. (A) Tumor cells invading the subcutaneous adipose tissue. (B) Tumor cells invading the skeletal muscle. (C) The horn cysts containing dense, laminated keratin. (D) The ductal structures lined by one or two layers of tumor cells, and containing eosinophilic materials. (E) The ductal structures, nests and strands of tumor cells embedded in a fibrosclerotic stroma (Hematoxylin-eosin, original magnifications: A × 100, B × 100; C × 100, D × 100, E × 200). MAC = microcystic adnexal carcinoma.

Histopathological examination showed a dermal-based neoplasm with infiltrative growth pattern and deep invasion of subcutaneous adipose tissue (Fig. 2A) and skeletal muscle (Fig. 2B). The tumor lacked any connection to the epidermis and consisted of horn cysts (Fig. 2C), ductal structures (Fig. 2D), nests, and strands of neoplastic cells embedded in a fibrosclerotic stroma (Fig. 2E). The horn cysts contained dense, laminated keratin. The ductal structures were lined by one or two layers of neoplastic cells and contained eosinophilic materials. No perineural invasion was detected. Cytologic atypia was minimal and no significant mitotic figures were identified.

The patient’s postoperative course continues to do well with good return of eyelid function, acceptable cosmetic outcome, and no evidence of disease at 12-year follow up. Informed consent was obtained from the patient for publication of this case report details.

### 3.2. High rates of misdiagnosis and local recurrence of the eyelid and orbital MAC

**Figure 3.**
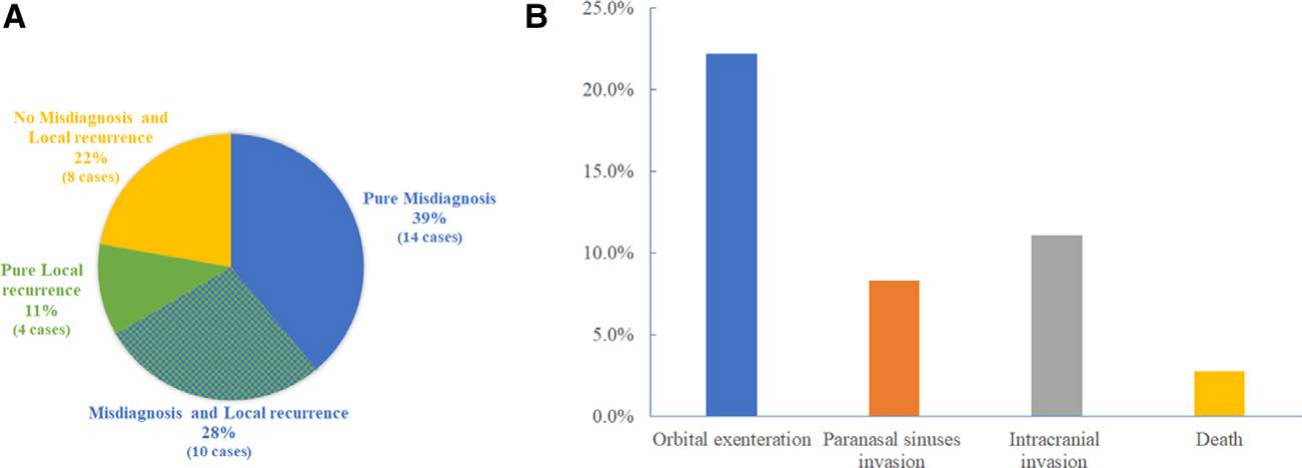
Misdiagnosis, local recurrence and serious complications of the eyelid and orbital MAC. (A) Misdiagnosis and local recurrence. (B) The serious complications. MAC = microcystic adnexal carcinoma.

The literature search revealed 36 cases of the eyelid and orbital MAC (Table 1), of which high rates of misdiagnosis and local recurrence are prominent features. Twenty-four (66.67%) of these cases were misdiagnosed at first visit, with basal cell carcinoma (BCC) and squamous cell carcinoma being the most common misdiagnosis in 7 cases each. Local recurrences were seen in 14 (38.89%) cases up to 4 times (Fig. 3A). Moreover, in 36 cases of the eyelid and orbital MAC, 5 cases originated from orbit, 19 cases were accompanied by perineural invasion.

**Table 1:** The characteristics and outcomes of 36 cases of the eyelid and orbital Microcystic adnexal carcinoma published in previous literature.

Author, yr	Age, Sex	Location	Tumor duration before diagnosis (mo)	Initial diagnosis	Perineural invasion	Initial treatment	Subsequent treatment	Recurrences	Metastasis	Follow-up (mo)
Khalil M, 1980^[29]^	70, M	Right upper eyelid	276	MAC	Yes	RA	OE	2	Right orbit, ethmoidal, sphenoidal, frontal and maxillary sinuses	3, died of other disease
Cooper PH, 1984^[24]^	51, F	Left nasal ala	13	Morphea-type BCC	Yes	Wide resection	MMS	3	Left orbit, left nasal turbinates, ethmoid sinus and orbital floor.	36
Glatt HJ, 1984^[30]^	20, F	Left lower eyelid	12	Syringoma	Yes	Excisional biopsy	Wide local excision	1	—	NM
	18, M	Right lower eyelid	24	MAC	Yes	Full thickness excision biopsy	Complete excision	1	Lacrimal punctum	9
Hesse RJ, 1995^[31]^	35, F	Right upper eyelid and eyebrow	NM	MAC	Yes	MMS	—	0	—	24
Hunts JH, 1995^[17]^	61, F	Left upper eyelid	24	MAC	NM	MMS	—	0	—	NM
Hoppenreijs VP, 1997^[32]^	50, F	Right lower eyelid	NM	MAC	Yes	Excision	Full thickness excision	4	—	NM
	83, F	Right lower eyelid	NM	SCC	Yes	Excisional biopsy	RA with rejection of OE	3	—	NM
	54, M	Right eyelid	36	SCC	Yes	Palliative therapy	—	0	Right orbit and frontal bone	NM
Brookes JL, 1998^[28]^	66, F	Left upper eyelid	NM	Chalazion	NM	Surgery	MMS	1	Lower punctum	NM
Esmaeli B, 1998^[33]^	48, M	Left upper eyelid	NM	Recurrent chalazion	NM	Surgical drainage	Resection	0	—	18
Duffy MT, 1999^[34]^	25, F	Left lower eyelid	NM	Epidermal acanthosis	Yes	Full thickness excision	MMS	0	—	96
	76, F	Right lower eyelid	NM	BCC	Yes	Full thickness excision	—	0	—	12
	70, F	Right lower eyelid	NM	BCC	Yes	Excision	—	0	—	NM
	95, F	Left lower eyelid	NM	BCC	NM	Full thickness excision	—	0	—	24
Ongenae KC, 2001^[18]^	43, F	Right eyelid	120	Trichoepithelioma	NM	MMS	—	0	—	36
Marshall J, 2003^[35]^	45, M	Left orbit	120	Fibrosclerosis	Yes	Excisional biopsy	OE&RA	0	—	6
Clement CI, 2005^[36]^	59, M	Left temporal	NM	SCC	Yes	MMS	MMS&RA	3	Left upper eyelid	71
	83, M	Right canthal	NM	SCC	NM	Resection	RA	1	Right upper eyelid	47
	73, F	Right upper eyelid	48	SCC	NM	Resection	MMS	4	—	91
Hasegawa S, 2005^[37]^	68, F	Right eyelid	3	MAC	NM	OE	—	0	Maxillary, supraorbital fissure and right skull base	24
Leibovitch I, 2006^[38]^	49, M	Medial canthal	<12	BCC	No	MMS	—	0	—	NM
	59, M	Medial canthal	<12	BCC	No	MMS	—	0	—	60
	62, M	Medial canthal	12-60	SCC	Yes	MMS	—	0	—	60
	31, F	Lower eyelid	12-60	MAC	No	MMS	—	0	—	60
	57, M	Medial canthal	12-60	BCC	No	MMS	—	0	—	NM
Nadiminti H, 2007^[39]^	54, F	Right upper eyelid	120	MAC	NM	MMS	MMS	1	—	5
Gomez-Maestra MJ, 2009^[40]^	75, F	Right orbit	27	SCC	Yes	MMS	OE&RA	3	Cavernous sinus and brainstem	51, died of intracranial invasion
Liyanage SE, 2010^[41]^	53, M	Left orbit	108	MAC	Yes	RA	—	NM	Cavernous sinus	NM
	24, F	Right lower eyelid	84	Chalazion	Yes	Intralesional steroids	OE&RA	1	Right orbit	NM
	70, M	Right lower eyelid	several years	MAC	NM	OE	—	NM	Right orbit	NM
Jeon S, 2010^[42]^	73, F	Left upper eyelid	8	Blepharitis	No	Oral antibiotics	MMS	0	—	24
Seaward JR, 2010^[43]^	53, M	Right eyebrow	NM	MAC	Yes	Wide local excision	—	0	—	14
Wu-Chen WY, 2011^[3]^	39, F	Left orbit	NM	Inflammation	NM	—	Rejection of OE	0	—	NM
Mukherjee B, 2018^[44]^	69, M	Left upper eyelid	36	MAC	NM	OE&RA	—	0	Orbital tissues and epidermis	6
Brent AJ, 2018^[45]^	29, M	Left orbit	4	Idiopathic orbital inflammation	Yes	Oral steroids	OE	1	Possible intracranial extension	144

& = and, BCC = basal cell carcinoma, F = female, M = male, MAC = microcystic adnexal carcinoma, MMS = Mohs micrographic surgery, NM = not mentioned, OE = orbital exenteration, RA = radiotherapy, SCC = squamous cell carcinoma.

### 3.3. Serious complications of the eyelid and orbital MAC

The serious complications of the eyelid and orbital MAC include disfigurement after orbital exenteration, invasion of paranasal sinuses or intracranial area, even death of the patient. Among the 36 cases reviewed in the literature search, 8 (22.22%) cases underwent orbital exenteration, 3 (8.33%) cases developed paranasal sinuses invasion, 4 (11.11%) cases experienced intracranial invasion and one of which died off (Fig. 3B).

## 4. Discussion

### 4.1. Oncogenesis

#### 4.1.1. Etiology.

The rarity of MAC makes it difficult to establish its etiology. However, ultraviolet light, radiation and immunosuppression may play a role in the pathogenesis of this tumor.^[8]^ MAC typically occurs almost on the head and neck, which are chronically exposed to ultraviolet light, and a slight advantage of left-side was reported. A series of cases in the United States show that 52% of the tumors are located on the left side of the face, the driver’s side (i.e., the side exposed to ultraviolet light) in this country.^[9]^ These researchers speculate that ultraviolet light may be the greatest risk factor for the development of MAC. Radiation was considered as another risk factor, because some cases of MAC occurred in sites previously treated with ionizing radiation (most commonly in acne).^[10]^ In addition, some cases of MAC were associated with immunosuppressive disease (such as kidney transplantation or chronic lymphocytic leukemia), which suggest the possible pathogenesis of immunosuppression on MAC.^[11,12]^

#### 4.1.2. Histopathology.

MAC was believed to originate from an adnexal keratinocyte capable of undergoing follicular or sweat gland differentiation.^[8]^ Histologically, MAC is characterized by horn cysts, ductal structures, nests and strands of basaloid epithelial cells, located intradermally but often infiltrating deeply into subcutaneous adipose tissue, skeletal muscle, nerves, fascia, and so on.^[13]^ Cytologic atypia was minimal and no significant mitotic figures were identified.^[14]^ MAC appears bland on histologic evaluation despite its locally aggressive behavior.^[15]^

The defining feature of MAC’s local growth pattern is that it typically extends a few centimeters beyond the clinically visible margins, and usually infiltrates deeply into the muscle, cartilage, bone, and nerves.^[6]^ MAC may invade adjacent tissue by expansion and infiltration, shelving or skating, and conduit spread. Shelving and skating represent the spread along fascial or capsular planes, muscle, galea, perichondrium, and periosteum. Conduit spread shows the tumor extends along the nerve.^[10]^ Shelving/skating and conduit spread often results in tissue invasion extending beyond the visible clinical margins of the tumor and also infiltrating the underlying subcutis or even deeper tissue.^[16]^

When MAC occurs in the eyelid or periocular regions, the tumor may spread to the paranasal sinuses or the cavernous sinus through foramen and fissures of the orbit, causing paranasal sinuses or intracranial metastasis.^[17,18]^

#### 4.1.3. Immunohistochemistry.

MAC does not have unique immunohistochemical characteristics, but epithelial membrane antigen (EMA), cytokeratin (CK) and carcinoembryonic antigen have been shown to be helpful in differentiating MAC from other tumors in histologic differentiation, highlighting eccrine and pilar differentiation.^[10]^ Particularly, EMA and CK immunostaining are the most reliable. EMA is a strong marker for ductal structures.^[8]^ Among the most commonly used CK markers, positive CK15 may help distinguish MAC from invasive BCC and squamous cell carcinoma.^[9]^ Carcinoembryonic antigen is considered to be a selective marker for demonstrating ductal differentiation in MAC, which is beneficial for eccrine and apocrine ducts in MAC.^[6]^ Ber-EP4, a monoclonal antibody, is believed to be useful for distinguishing between MAC (Ber-EP4 negative) and BCC (Ber-EP4 positive).^[9]^

Although these helpful immunohistochemical studies are available, they are rarely needed to diagnose MAC. The gold standard remains an adequately deep biopsy specimen evaluated by a dermatopathologist with light microscopy of hematoxylin and eosin-stained sections and appropriate clinicopathologic correlation.^[15]^

#### 4.1.4. Molecular features.

The molecular pathogenesis of MAC is not fully understood, but recent studies have identified several genetic alterations that may contribute to its development and progression. Mutations in the tumor suppressor gene *TP53* and activation of the JAK/STAT signaling pathway have been reported in some cases of MAC, suggesting a role for dysregulated cell proliferation and apoptosis.^[19]^ Other potential molecular targets for MAC include the epidermal growth factor receptor, which is overexpressed in some cases and may suggest a potential target for therapy.^[19,20]^

### 4.2. Clinical presentation

#### 4.2.1. Region.

**Figure 4.**
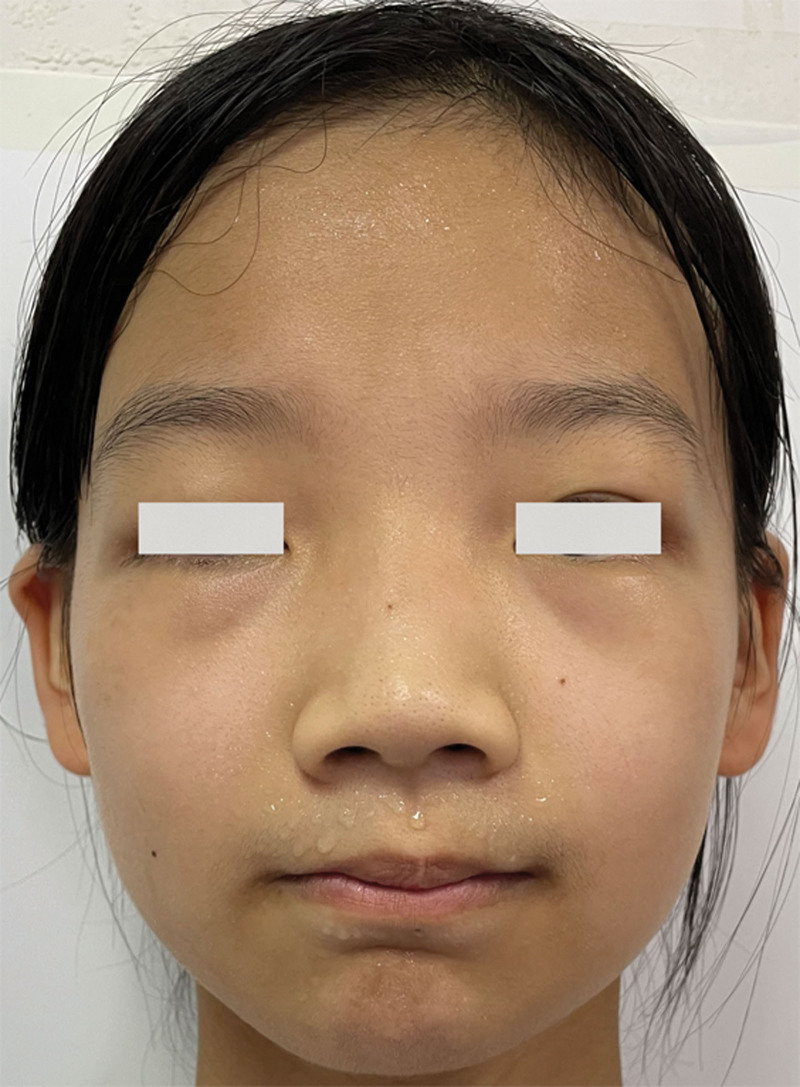
The upper lip, nasolabial folds and forehead containing abundant sweat glands. MAC typically arises from these three regions. MAC = microcystic adnexal carcinoma.

MAC typically arises from the head and neck region, most commonly on the upper lip, nasolabial folds, and forehead.^[8]^ These 3 regions contain abundant sweat glands (Fig. 4). However, rare cases of MAC have been reported in the eyelid and orbit (Table 2), which can present a diagnostic and therapeutic challenge due to their anatomical complexity and functional significance.

**Table 2:** Clinical presentation of the eyelid and orbital Microcystic adnexal carcinoma.

Location	Symptoms	Signs
Eyelid	no symptom; numbness, burning, tingling, and other sensations.	slow-growing, firm, flesh-colored to yellowish nodule or plaque often with ill-defined margins, and overlying telangiectasia
Orbit	no symptom; numbness, burning, tingling, and other sensations.	deformity, ptosis, enophthalmos, limitation of eye movement, diplopia or visual impairment

#### 4.2.2. Symptoms and signs.

Clinical presentation of MAC of the eyelid is not specific, but usually presents as an asymptomatic, slow-growing, firm, flesh-colored to yellowish nodule, plaque often with ill-defined margins, and overlying telangiectasia, which is thought to be benign.^[7]^ If perineural invasion of MAC occurs, patients may experience numbness, burning, tingling, and other sensations. When MAC occurs in orbit, some special clinical manifestations, such as deformity, ptosis, enophthalmos, limitation of movement, diplopia or visual impairment, may be seen, but it is indistinguishable from benign lesions.^[3]^

### 4.3. Management

The management of MAC of the eyelid and orbit depends on several factors, including the size and location of the tumor, as well as the patient’s age, comorbidities, and preferences. When orbital MAC spreads to the paranasal sinuses or the cavernous sinus, an interprofessional team is needed to evaluate and treat patients.

#### 4.3.1. Surgery.

Surgical excision is the most definitive method of treatment of MAC, including MMS and wide local excision.^[7]^ MMS involves horizontal en-face sections of the entire outer surface of the excised tissue, thereby examining 100% of the peripheral and deep margins.^[10]^ In the literature, wide local excision had a recurrence rate of 54.5%, whereas MMS had a recurrence rate of 6.0%.^[21,22]^ Based on the local aggressiveness of MAC, MMS is considered the gold standard of treatment for this tumor. MMS allows tissue conservation while providing increased attention to tumor margins and the highest possibility of long-term cure.^[23]^

#### 4.3.2. Radiotherapy.

Radiotherapy may be considered in cases with high-risk features or incomplete resection, and is often used as an adjuvant therapy in eyelid and orbital MAC to improve life quality of patient.^[24]^

#### 4.3.3. Chemotherapy.

In cases of advanced or metastatic MAC, systemic chemotherapy or targeted therapy may provide palliative benefit, but the efficacy of these approaches is limited and further research is needed to identify more effective treatments. There have been reports of chemoradiation with carboplatin/paclitaxel combination used in a patient refusing surgical management that remained tumor-free for 6 years.^[25]^

### 4.4. Misdiagnosis, local recurrence and serious complications of the eyelid and orbital MAC

#### 4.4.1. Misdiagnosis.

Due to its rarity and nonspecific clinical and histopathological presentation, MAC of the eyelid and orbit can often be misdiagnosed or overlooked, leading to delayed diagnosis and suboptimal management.^[26]^ Clinically, MAC of the eyelid and orbit typically presents as a slow-growing, painless nodule or plaque that may be mistaken for other benign lesion, such as chalazion, papilloma, or syringoma.^[17]^ In order to reduce or avoid the misdiagnosis, it is important to increasing the awareness of physician for MAC. Histopathologically, superficial areas of MAC may resemble syringoma, desmoplastic trichoepithelioma, trichoadenoma, or morpheaform BCC.^[21]^ Thus, a superficial biopsy leads to misdiagnosis in up to 30% of cases, highlighting the importance of obtaining a full thickness biopsy specimen.^[15]^

#### 4.4.2. Local recurrence.

MAC has a tendency for local recurrence because of its locally extensive invasive growth pattern. The Surveillance, Epidemiology and End Results database analysis showed that out of 223 reported cases of MAC, the local recurrence rate after any surgery was 18%.^[1]^ In our literature research, local recurrences of the eyelid and orbital MAC were seen in 38.89% cases, up to four times.

Based on the infiltrative growth nature of MAC, an inadequate excision may result in a higher risk of persistent tumor at the surgical margins, leading to a higher risk of recurrence.^[27]^ Therefore, complete excision with clear margin is necessary.

Long-term follow-up of the patient with MAC is another important factor against the high rate of local recurrence.^[8]^ Clinical monitoring involves skin examination with palpation of the surgical scar and the draining nodal basin. It is suggested that clinical examination should be performed every 6 to 12 months during the first 5 years of follow-up.^[22]^ Afterward, it may be appropriate to reduce examination frequency. MAC patients should be followed clinically indefinitely.^[15]^

#### 4.4.3. Serious complications.

MAC is characterized by low-grade malignancy, but when it occurs in the eyelid or orbit, the tumor can spread to the paranasal sinuses or the cavernous sinus, leading to serious complications such as disfigurement after orbital exenteration, invasion of the paranasal sinuses or intracranial area, even death of the patient.^[28]^ Orbital exenteration and disfigurement will have a huge psychological impact on the patient.^[3]^ To prevent these serious complications, early and accurate diagnosis, complete excision, and long-term follow-up are essential. Moreover, an interprofessional team consisting of ophthalmologist, otolaryngologist, neurologist, dermatologist, pathologist, radiologist is needed to evaluate and treat the patients of MAC.

## 5. Conclusion

MAC is a rare, but commonly misdiagnosed, frequently recurrent malignancy, which poses a significant diagnostic and treatment challenge to clinicians. Although primary eyelid or orbital MAC is rare, it can lead to serious complications, including disfigurement after orbital exenteration, invasion of the paranasal sinuses or intracranial area, and even death. MAC of the eyelid and orbit requires a multidisciplinary approach to diagnosis and management. The present study emphasizes the importance of increasing awareness, early and accurate diagnosis, complete excision, long-term follow-up, and interprofessional team for management of the eyelid and orbital MAC.

## Author contributions

**Conceptualization:** Yaru Dong.

**Data curation:** Shiwei Huang, Yang Xia, Yueyang Zhu, Zhiyuan Ren, Yaru Dong.

**Formal analysis:** Shiwei Huang.

**Funding acquisition:** Yaru Dong.

**Investigation:** Shiwei Huang, Yang Xia, Yueyang Zhu.

**Methodology:** Yang Xia, Yueyang Zhu.

**Project administration:** Yueyang Zhu.

**Supervision:** Yaru Dong.

**Validation:** Yaru Dong.

**Visualization:** Zhiyuan Ren.

**Writing – original draft:** Shiwei Huang.

**Writing – review & editing:** Yueyang Zhu, Zhiyuan Ren, Yaru Dong.
